# Effectiveness of mesenchymal stem cell therapy in cats with chronic gingivostomatitis

**DOI:** 10.18849/ve.v9i1.680

**Published:** 2024-02-07

**Authors:** Omid Nekouei+, San Tung Wong+, Tiffany Ka Yi Leung+, Qi An Ngai+, Wing Sum Wong+, Angel Almendros+

**Keywords:** CATS, FELINE, FELINE CHRONIC GINGIVOSTOMATITIS, REFRACTORY, STEM CELL, TREATMENT

## Abstract

**PICO question:**

In cats with chronic gingivostomatitis, does using intravenous mesenchymal stem cell therapy, compared to not using it, lead to the improvement of clinical signs?

**Clinical bottom line:**

**Category of research:**

Treatment.

**Number and type of study designs reviewed:**

Five interventional studies (clinical trials).

**Strength of evidence:**

Moderate.

**Outcomes reported:**

The reviewed studies indicate intravenous mesenchymal stem cell (MSC) therapy is safe to administer and can be effective in remission or alleviating the clinical signs of refractory, chronic gingivostomatitis in cats that have undergone full-mouth tooth extraction.

**Conclusion:**

The collective evidence supports the intravenous administration of mesenchymal stem cell (MSC) therapy in cats with chronic gingivostomatitis following dental extraction. However, conducted clinical trials are prone to different degrees of bias due to the lack of independent control groups, the small number of subjects, and enrolling subjects with various severity of the disease. Therefore, more robust evidence can be obtained through well-designed randomised controlled trials to confirm the observed positive effects of the treatment in cats with a broader range of characteristics.

## How to apply this evidence in practice

The application of evidence into practice should take into account multiple factors, not limited to: individual clinical expertise, patient’s circumstances and owners’ values, country, location or clinic where you work, the individual case in front of you, the availability of therapies and resources.

Knowledge Summaries are a resource to help reinforce or inform decision-making. They do not override the responsibility or judgement of the practitioner to do what is best for the animal in their care.

## Clinical scenario

Six months ago, you diagnosed a seven-year-old female domestic short-haired (DSH) cat with severe gingivostomatitis based on the presence of consistent clinical signs, a thorough oral examination, a complete blood work, and oral radiographs. You performed a full-mouth tooth extraction and administered supportive treatment, but the clinical signs persist, and the cat still suffers from pain and has not gained weight. Current treatment modalities, such as oral or injectable corticosteroids and oral cyclosporin were declined by the owner due to concerns of side effects in the unwell animal. You are now looking for alternative therapy options to improve the quality of this cat's life and the remission of clinical signs. A colleague suggests intravenous mesenchymal stem cell (MSC) therapy; however, you are uncertain about the clinical efficacy and safety of this option due to the limited evidence available.

## The evidence

Following the eligibility criteria (see Methodology Section), five interventional studies were deemed relevant to the PICO question. Four clinical trials investigated the efficacy and safety of treating cats with feline chronic gingivostomatitis (FCGS) with autologous or allogeneic adipose-derived MSCs (Arzi et al., 2016; 2017; 2020; and 2021), and the other study included treatment with placenta-derived MSCs (Febre et al., 2022). Positive clinical responses to MSC therapy were found in all studies where cats had undergone previous full-mouth tooth extraction (Arzi et al., 2016; 2017; 2020; and Febre et al., 2022). However, in one study, MSC therapy appeared to lack efficacy when applied to affected cats prior to teeth extraction (Arzi et al., 2021). The appraised evidence moderately demonstrated that MSC therapy was safe and resulted in notable clinical improvement in most cats that did not respond to full-mouth tooth extractions. However, these studies had some limitations and were prone to different degrees of bias. The lack of an independent control group (consequently, no randomisation and blinding) and the small unjustified sample sizes (some labeled as pilots) reduced the robustness and generalisability of the findings. Spontaneous recovery from FCGS has never been reported, and cats that did not respond to medical interventions are often euthanised due to the debilitating nature of the disease, which can add to the value of the findings. All in all, our Knowledge Summary provides moderate support to MSC therapy to improve the clinical signs of FCGS-affected cats.

## Summary of the evidence

**Arzi et al. (2016):** 

Population	Client-owned cats, 3–14.5 years old, presented with refractory feline chronic gingivostomatitis (FCGS). They had no other primary comorbidities, and did not respond to full-mouth tooth extraction performed at least 6 months before enrollment.
Sample Size	9 cats.
Intervention Details	If corticosteroid or other immunosuppressive therapy was prescribed, it had to be discontinued 2 weeks prior to and for the entire duration of the trial. Subcutaneous abdominal fat was collected from recruited cats to obtain autologous stem cells. Intravenous fluid administration (lactated Ringer’s solution) was initiated at least 30 minutes before treatment. A single dose (2 mg/kg) of diphenhydramine was administered subcutaneously 20 minutes before treatment. Each cat received two intravenous transfusions of 20 million (5 million stem cells per kg) fresh autologous stem cells, 1 month apart. Each dose of 20 million stem cells was administered slowly over a period of 20–30 minutes. All cats were hospitalised for 48–72 hours after transfusion to monitor for adverse reactions. Cats were evaluated before and at 1, 3, and 6 months after treatment. A final examination was performed at 6 months after the first stem cell transfusion.
Study design	Clinical trial (uncontrolled).
Outcome studied	Haematological and immunomodulatory variables. Complete blood count (CBC). Serum biochemistry. Serum protein. Blood lymphocyte. Severity of oral inflammatory lesions. Neutrophil count. Cytokine analyses (Interferon-γ, tumour necrosis factor-α, interleukin-1β, IL-6). CD3 and CD20 lymphocytes. % of circulating CD8+ T cells. % of CD8^lo ^cells. % of circulating CD4+ T cells. CD4/CD8 ratio. Total serum globulin level. Anti-bovine serum albumin levels. Oral biopsies. Stomatitis disease activity index (SDAI). Appetite, activity level, grooming behaviour, and perceived oral comfort. Severity of oral inflammatory lesions.
Main findings (relevant to PICO question)	7/9 cats completed the study, of which five responded to treatment within 1–3 months of the first stem cell administration. Three with complete clinical remission. Two with substantial clinical improvement. Two cats had minimal or no clinical response. 2/9 cats discontinued the trial within the first 2 months of the study because of the owner’s decision to administer corticosteroids to them. The responder cats began eating more, gaining weight, resumed grooming behaviour and sociability. Intravenous infusion of a relatively high dose (20 million cells per cat) of autologous adult stem cells was safe and well-tolerated. The treatment showed immunomodulatory effects in responder cats, including: Reduction in CD8+ T cells. Reduced systemic inflammatory response, including reduced levels of circulating neutrophils and serum IFN-γ and IL-1β. Elevation in serum IL-6 concentration. The three cats that responded with complete clinical remission showed a sustained increase in TNF-α. Response to stem cell therapy was often delayed by 2–4 months after the first injection. Only a transient transfusion reaction was observed in the cats.
Limitations	The SDAI upon entry was different among the subjects. Small, unjustified sample size. Lack of an independent control group (consequently, no randomisation and blinding). Post-treatment oral biopsies were only available for three cats. Limited generalisability (not accounting for potential confounders).

**Arzi et al. (2017):** 

Population	Client-owned cats, 3–12 years old, presented with refractory feline chronic gingivostomatitis (FCGS). All cats underwent full-mouth tooth extraction at least 6 months prior to enrollment and did not respond to the treatments.
Sample Size	7 cats.
Intervention Details	If corticosteroid or other immunosuppressive therapy had been prescribed, it was discontinued two weeks prior to and for the entire duration of the clinical trial. Two intravenous transfusions of 20 million (5 million stem cells per kg) fresh allogeneic adipose-derived stem cells were administered one month. The treatment was administered over a period of 20–30 minutes by dividing the total dose into four separate aliquots (5 million cells at a time). All cats were hospitalised for 24 hours post-treatment to monitor for adverse reactions. All cats were evaluated before treatment and at 3 and 6 months post-treatment. A final examination was performed at 6 months after the first treatment. However, cats were followed up continuously and data were presented from the final recheck as well.
Study design	Clinical trial (uncontrolled).
Outcome studied	Haematological and immunomodulatory variables. Complete blood count (CBC). Serum biochemistry profile. Blood lymphocyte phenotype. % of CD4+ T cells. % of CD8+ T cells. % of CD8^lo ^cells. CD4/CD8 ratio. Globulin concentration. Neutrophil number. Serum IFNγ concentration. Serum TNFα concentration. Serum IL-6 concentration. Lymphocyte proliferative ability. Histopathology on oral biopsies. Stomatitis disease activity index (SDAI). Body weight, appetite, activity level, grooming behaviour, sociability, and perceived oral comfort. Lesion severity.
Main findings (relevant to PICO question)	4/7 cats responded positively to the treatment at the formal end of study (6 months). Two cats with substantial clinical improvement. Two cats with complete remission within 18–20 months. The other 3/7 cats had either minimal or no clinical response. Responder cats (4/7) began eating more, gaining weight, resuming grooming behaviour and sociability. Improvement of oral mucosal lesions was observed in the responders (4/7 cats). Cats that did not respond to treatment had higher initial neutrophil counts, globulin concentrations, and serum IFNγ concentrations compared to responder cats. 2/3 cats that did not respond to treatment had detectable systemic TNFα concentrations before treatment. The treatment was deemed safe to administer, and no side effects were observed.
Limitations	The stomatitis disease activity index (SDAI) upon entry was different among the subjects. Small, unjustified sample size. Lack of an independent control group (consequently, no randomisation and blinding). Oral biopsies post treatment were only available for 3/7 cats. Limited generalisability (not accounting for potential confounders).

**Arzi et al. (2020):** 

Population	Client-owned cats, 3–11 years old, presented with refractory feline chronic gingivostomatitis (FCGS) to four institutions (University of California Davis, Cornell University, and two private dental specialty practices). Recruited cats had no other primary comorbidities and did not respond to full-mouth extractions performed at least 6 months prior to enrollment.
Sample Size	18 cats.
Intervention Details	All systemic or topical corticosteroids or other immunosuppressive therapies were discontinued two weeks prior to and for the entire duration of the trial. Six cats acted as their own control for 6 months prior to treatment. Two intravenous transfusions of 20 million (5 million stem cells per kg) adipose-derived mesenchymal stem cells (ASCs) were administered 1 month apart. The stem cells cryopreserved and revived in culture for 72 hours prior to administration: Five cats received allogeneic ASCs derived from two separate specific pathogen-free cats. 13 cats received autologous ASCs initially but switched to allogeneic ASCs. The total dose was divided into four separate aliquots (5 million cells at a time) and administered over a period of 20–60 minutes within 24 hours of processing. All cats were hospitalised for 24 hours post-treatment to monitor for adverse effects. Cats were evaluated until 6 months post-treatment (except for one that was lost to follow-up after 3 months). Cats were evaluated at day 0 and at 1 month (second injection), 3 months and 6 months after the first ASC treatment (study exit). However, cats were followed up continually for up to 24 months and data presented include the final recheck as well.
Study design	Clinical trial (partial historical control).
Outcome studied	Haematological and immunomodulatory variables. Complete blood count (CBC). Serum biochemistry profile. Blood lymphocyte phenotype. % of CD8+ T cells. % of CD8^lo ^cells. CD4/CD8 cells. Globulin concentration. Neutrophil number. Lymphocyte proliferative ability. Histopathology on oral biopsies. Stomatitis disease activity index (SDAI). Body weight, appetite, activity level, grooming behaviour, sociability, and perceived oral comfort. Lesion severity.
Main findings (relevant to PICO question)	None of the six self-control cats showed clinical improvement without the treatment. 13/18 cats had a positive response to treatment: 5/13 were completely recovered. 8/13 had marked resolution of clinical signs. 5/18 cats did not respond to the treatment. 13 responder cats had decreasing trend of globulin concentration over time and a lower % of CD8^lo^ cells before therapy. 13 responder cats gained weight and returned to normal eating behaviour, activity levels, grooming, and sociability. Substantial reduction in oral mucosal inflammation was observed in the 13 responder cats. Adverse effects of treatment included oedema in the forelimb used for IV administration, skin necrosis, increased respiratory rate, vomiting, and diarrhea, which were non-life threatening and mostly resolved. Clinical response to treatment took 3–6 months. Remission or clinical improvements were permanent in the 13 responder cats.
Limitations	The SDAI upon entry was different among the subjects. Lack of an independent/parallel control group (consequently, no randomisation and blinding) – only 6/18 FCGS cats served as their own controls. Limited generalisability (not accounting for potential confounders).

**Arzi et al. (2021):** 

Population	Client-owned cats, 1–13 years old, presented with feline chronic gingivostomatitis (FCGS). Recruited cats had painful FCGS with no other underlying health conditions and did not undergo full-mouth tooth extraction.
Sample Size	5 cats.
Intervention Details	The cats were free of corticosteroids and/or antibiotic treatment for at least 14 days prior to the trial. Pain treatment with opioids was continued as needed. Two intravenous transfusions of 20 million (5 million stem cells per kg) adipose-derived mesenchymal stem cells (ASCs) were administered 1 month apart. 4/5 cats received allogeneic ASCs obtained from specific pathogen-free cats. 1/5 cat received autologous ASCs obtained from the subcutaneous abdominal fat of the same cat. Treatment was administered over 20–40 minutes by dividing the total dose into four separate aliquots (5 million cells at a time). All cats were hospitalised for 24 hours post-treatment to monitor for adverse reactions. All cats were evaluated prior to and at 6 months post-treatment.
Study design	Clinical trial (uncontrolled).
Outcome studied	Haematologic and biochemical profile. White blood cell count. CD4:CD8 T cell ratio. % of CD8^lo^ T cells. Polymorphonuclear leucocyte count. Albumin and globulin concentration. Bodyweight, appetite, sociability, and grooming behvaiours. Oral mucosal lesions. Stomatitis disease activity index (SDAI).
Main findings (relevant to PICO question)	4/5 cats completed the study, and one left the study 3 months after treatment. 3/5 cats did not display any clinical improvement. 2/5 cats exhibited mild clinical improvement. None of the cats exhibited immune modulation responses. 3/5 cats had sustained elevation of CD8+ T cells and decreased CD4:CD8 ratio prior to treatment and did not improve at the exit examination. Variable leucocytosis due to neutrophilia and a tendency for hyperglobulinemia. Absence of normalisation of the CD4:CD8 ratio. None of the cats exhibited any adverse events during administration and throughout the study.
Limitations	The SDAI upon entry was different among the subjects. Small, unjustified sample size. Lack of an independent control group (consequently, no randomisation and blinding). Limited generalisability (not accounting for potential confounders, such as viral infection status).

**Febre et al. (2022):** 

Population	Client-owned cats, 3–11 years old, presented with refractory feline chronic gingivostomatitis (FCGS) to three veterinary clinics. Recruited cats had persistent clinical signs and inflammatory lesions specific to FCGS and did not respond to a sub-total or total dental extraction or supportive treatment with cyclosporine, non-steroidal anti-inflammatories, or interferon-omega for at least 2–3 months before enrollment.
Sample Size	8 cats.
Intervention Details	Corticosteroids were discontinued 2 weeks before inclusion and not allowed during the study. Opioid analgesics, gabapentin and nonsteroidal anti-inflammatory drugs (NSAIDs) were accepted if the animals were already under such medication. All cats received a single intravenous infusion of 10 million viable feline placenta-derived mesenchymal stromal cells that were cryopreserved and thawed the day before injection with supplementation of lactated Ringer’s solutions and heparin sulfate 200 IU. Treatment was administered over a period of 20–30 minutes. Follow-up evaluations on cats were conducted at 15 days, 2, 3, and 6 months post-treatment.
Study design	Clinical trial (uncontrolled).
Outcome studied	Pro-inflammatory clinical response. Oral lesion severity. Appetite, activity level, grooming behaviour, perceived oral comfort. Stomatitis disease activity index (SDAI). Respiratory and heart rates during infusion and before discharge.
Main findings (relevant to PICO question)	All eight cats attended follow-up visits except one cat at one appointment. All eight cats showed clinical improvement of varying degrees and kinetics in response at the 6-month follow-up evaluation. 4/8 cats showed good improvement (50% reduction in SDAI score). 2/8 cats displayed moderate improvement (30-50% reduction in SDAI score). 2/8 cats showed mild improvement (12-30% reduction in SDAI score). The median of SDAI score of the cats decreased from 6 to 8 from the inclusion point to the 6-month of evaluation. Reduction in oral mucosal lesions was observed. Vomiting was occasionally observed following medetomidine treatment and was reported in one cat during infusion under intubation. No adverse events were recorded at 2, 3, and 6 months follow-up visits, except for one cat that exhibited worsening inflammatory signs on day 15.
Limitations	The SDAI upon entry was different among the subjects. Small, unjustified sample size. Lack of an independent control group (consequently, no randomisation and blinding). Limited generalisability (not accounting for potential confounders, such as viral infection status).

## Appraisal, application and reflection

Feline chronic gingivostomatitis (FCGS) is a serious, immune-mediated, oral mucosal inflammatory disease in cats. While the majority of cats respond to total or sub-total dental extraction as the current standard treatment, approximately 30% of cats do not improve (Jennings et al., 2015). Palliative treatment before and/or after dental extraction includes the use of corticosteroids, interferon omega and cyclosporin for their anti-inflammatory and immunomodulatory effects (Hennet et al., 2011; and Lommer, 2013). However, these options often come with several side effects, such as immune suppression due to corticosteroids and gastrointestinal upset for interferon omega and cyclosporin. Mesenchymal stem cell (MSC) therapy is an emerging approach to treating cats with immune-mediated inflammatory disorders, such as FCGS. Mesenchymal stem cells have been used in several conditions where immunomodulation was intended, such as chronic kidney disease in cats (Vidane et al., 2017) and inflammatory bowel disease in dogs (Pérez-Merino et al., 2015). Additionally, other studies have assessed its efficacy and safety in dogs with periodontal disease (Inukai et al., 2013) and osteoarthritis (Huňáková et al., 2020), where no complications were reported. For FCGS, the available evidence on the safety and efficacy of MSC therapy in clinical settings is still scarce. This knowledge summary was prepared to address a critical question posed by feline practitioners regarding the efficacy of intravenous MSC therapy in cats with FCGS.

Following the eligibility criteria, five interventional studies were found relevant to the PICO question. Of which, four studies provided support for the safety and efficacy of MSC therapy toward complete remission or substantial improvement in the clinical signs of FCGS in cats that had undergone full-mouth tooth extraction (Arzi et al., 2016; 2017; 2020; and Febre et al., 2022). However, one trial indicated that MSC therapy prior to full-mouth dental extraction did not lead to substantial improvement (Arzi et al., 2021). Four studies investigated the treatment with two successive intravenous (IV) infusions of adult autologous or allogeneic MSCs (Arzi et al., 2016; 2017; 2020; and 2021), and one study applied a single IV infusion of placenta-derived MSCs (Febre et al., 2022). These five studies shared several outcome parameters in determining the efficacy of the treatment, including improvement in oral lesions, body weight, and behaviour of the subjects.

Although all five studies were clinical trials, they could not collectively provide a strong level of evidence in support of MSC therapy for FCGS in cats due to a number of limitations in their design and conduct. One of the key design issues was the lack of an independent control group of subjects to compare the treatment effect with untreated or other types of standard medical interventions in parallel. Therefore, all of these trials lacked randomisation and blinding, which are the most important components of good clinical trials. This issue makes the reviewed studies prone to different levels of bias. However, with respect to the progressive nature of refractory FCGC and the fact that spontaneous recovery has not been reported, these studies can still provide a moderate level of evidence in support of the treatment.

There were no sample size calculations or justifications in the reviewed studies. Four studies (Arzi et al., 2016;  2017;  2021; and Febre et al., 2022) had a small number of cats, mostly considered pilots. Therefore, several potential confounding factors, such as age, sex, weight, breed, and severity or stage of the disease, could not be considered. This can further limit the generalisability of the results with respect to the broad spectrum of factors affecting FCGS progress and complications in natural circumstances. For instance, concurrent diseases were not determined in these studies, which might have affected the clinical course of the disease and response to MSC therapy. Arzi et al. (2021) reported that the concurrent development of periodontitis in cats might have had a confounding effect on studying the clinical efficacy of MSC therapy. Moreover, the stomatitis disease activity index (SDAI) for each subject differed upon entry into the studies, further complicating the interpretations of their results. It is noteworthy that the study by Febre et al. (2022) was funded by Vetbiobank. Four of the authors were employees of Vetbiobank, and one author was a principal shareholder, which was declared in the conflict of interest statement.

From the five studies, intravenous autologous and allogeneic MSC therapy appear well-tolerated by cats with FCGS. Most side effects observed were mild to moderate, including transfusion reactions, increased respiratory rate, vomiting, and diarrhea which were resolved either spontaneously or therapeutically with no further complications (Arzi et al., 2016; and 2020). Skin necrosis requiring a skin graft was recorded only in one cat that developed oedema at the injection site (Arzi et al., 2020). Nevertheless, the small sample sizes and variable follow-up periods (6–24 months) hindered the long-term evaluations of the safety of MSC therapy in treating FCGS in cats. The potential detrimental consequences of stem cell therapy (e.g., tumorigenesis) have been documented in human patients (Bauer et al., 2018).

The reviewed studies recommended two IV transfusions of 20 million MSCs, 1 month apart, following a lack of response to full-mouth or premolar-molar tooth extraction (Arzi et al., 2016; 2017; 2020; and 2021). Although it was generally demonstrated that both intravenous adipose-derived autologous and allogeneic MSCs were safe and efficacious for treating refractory FCGS, autologous MSCs might be preferred due to a faster response period and higher efficacy (Arzi et al., 2017; and 2021). An infusion rate of 2 million cells per minute was recommended to eliminate potential transfusion reactions (Arzi et al., 2016). Furthermore, a useful biomarker was suggested for predicting the response to adult MSC therapy, i.e., decreased CD8^lo^ (%) within the CD8+ T cells (Arzi et al., 2016; and 2020). Only one study investigated placenta-derived MSCs with a lower dose of 10 million cells and a single IV transfusion, which showed clinical efficacy despite the abovementioned limitations (Febre et al., 2022). One limitation in the present Knowledge Summary is the fact that 4/5 studies happened to be conducted by one group of researchers; therefore, the paucity of comparable evidence from other places regarding our PICO should be considered in using this summary. A recent descriptive survey conducted by the same research group (Soltero-Rivera et al., 2023) evaluated the long-term safety and efficacy of the intravenous MSC treatment of 38 FCGS patients, including some of the cases included in ‘Arzi et al., clinical trials’ (summarised here) and no adverse events were noted during the 2–9 years of follow-up. The results of this survey further lend support to using both autologous and allogeneic MSC as an efficacious and safe therapeutic option for refractory FCGS. We did not include the latter survey as an independent study in our current knowledge summary for multiple reasons: 1) not meeting one of our important inclusion criteria (i.e., ‘presented adequate details on the intervention’), 2) being at the lowest levels of the pyramid of evidence (as a descriptive survey), and 3) overlapping of cases with the trial subjects already included in our summary.

In conclusion, available studies collectively provide a moderate strength of evidence in support of using intravenous MSCs to treat cats with FCGS refractory to sub-total to total dental extraction. To enhance the strength of evidence and eliminate potential sources of bias, conducting randomised, double-blinded, controlled trials with a sufficient number of subjects is recommended. Nonetheless, the ethical considerations and complications of conducting such clinical trials are recognised.

## Methodology

**Search strategy:** 

Search terms	CAB Abstracts on EBSCOhost: From 1973 to week 19, 2023 PubMed via NCBl: From 1910 to week 19, 2023
Dates searches performed:	CAB Abstracts: ((cat* OR feline) AND (gingivostomatitis OR chronic gingivostomatitis OR refractory gingivostomatitis OR "FCGS")) AND ("mesenchymal stem cell*" OR "stem cell*" OR "MSC*") PubMed: ((cat* OR feline) AND (gingivostomatitis OR chronic gingivostomatitis OR refractory gingivostomatitis OR “FCGS”)) AND (mesenchymal* OR stem cell OR MSC* OR mesenchymal stromal cell*)
Search terms	9 May 2023

**Exclusion / inclusion criteria:** 

Exclusion	Unrelated to PICO. *In vitro *studies. Single case reports. Reviews.
Inclusion	Studies related to PICO in English. *In vivo *studies. Peer-reviewed original articles. Presented adequate details on the intervention.

**Search outcome:** 

Database	Number of results	Excluded –Unrelated to PICO question	Excluded – In vitro	Excluded – Single case report	Excluded – Review	Total relevant papers
CAB Abstracts	13	8	1	1	2	1
PubMed	12	5	1	0	1	5
Total relevant papers when duplicates removed						5

## Author contributions

**Omid Nekouei:** Conceptualisation, Methodology (lead), Supervision, Validation, Formal analysis, Writing – Review & editing. **San Tung Wong:** Data curation, Formal analysis, Writing – Original draft preparation, Methodology. **Tiffany Ka Yi Leung: **Data curation, Formal analysis, Writing – Original draft preparation, Methodology. **Qi An Ngai:** Data curation, Formal analysis, Writing – Original draft preparation, Methodology. **Wing Sum Wong:** Data curation, Formal analysis, Writing – Original draft preparation, Methodology. **Angel Almendros:** Conceptualisation, Validation, Formal analysis, Writing – Review & editing.

## ORCiD

Omid Nekouei: https://orcid.org/0000-0002-2427-4159

Angel Almendros: https://orcid.org/0000-0002-2441-7850

## Conflict of interest

The authors declare no conflicts of interest.
